# Terreneuvian Orthothecid (Hyolitha) Digestive Tracts from Northern Montagne Noire, France; Taphonomic, Ontogenetic and Phylogenetic Implications

**DOI:** 10.1371/journal.pone.0088583

**Published:** 2014-02-12

**Authors:** Léa Devaere, Sébastien Clausen, J. Javier Álvaro, John S. Peel, Daniel Vachard

**Affiliations:** 1 UMR 8217 Géosystèmes CNRS – Université Lille 1Villeneuve d’Ascq, France; 2 Centro de Astrobiología, Instituto Nacional de Técnica Aeroespacial, Consejo Superior de Investigaciones Científicas, Torrejón de Ardoz, Spain; 3 Department of Earth Sciences (Palaeobiology), Uppsala University, Uppsala, Sweden; University of Münster, Germany

## Abstract

More than 285 specimens of *Conotheca subcurvata* with three-dimensionally preserved digestive tracts were recovered from the Terreneuvian (early Cambrian) Heraultia Limestone of the northern Montagne Noire, southern France. They represent one of the oldest occurrences of such preserved guts. The newly discovered operculum of some complete specimens provides additional data allowing emendation of the species diagnosis. Infestation of the U-shaped digestive tracts by smooth uniseriate, branching to anastomosing filaments along with isolated botryoidal coccoids attests to their early, microbially mediated phosphatisation. Apart from taphonomic deformation, *C. subcurvata* exhibits three different configurations of the digestive tract: (1) anal tube and gut parallel, straight to slightly undulating; (2) anal tube straight and loosely folded gut; and (3) anal tube straight and gut straight with local zigzag folds. The arrangement of the digestive tracts and its correlation with the mean apertural diameter of the specimens are interpreted as ontogenetically dependent. The simple U-shaped gut, usually considered as characteristic of the Hyolithida, developed in earlier stages of *C. subcurvata*, whereas the more complex orthothecid type-3 only appears in largest specimens. This growth pattern suggests a distinct phylogenetic relationship between these two hyolith orders through heterochronic processes.

## Introduction

### Background

Hyoliths are fossil, marine, benthic organisms characterised by a conical calcareous conch closed by an operculum. They are restricted to the Palaeozoic and are particularly common and abundant in Cambrian strata. Despite their well-studied fossil record, their taxonomic and phylogenetic positions are still debated. They have been considered to be molluscs [1]–[10] but other authors [11]–[14] suggested they comprised a distinct phylum (see also [15]). Herein, we take a neutral stance and regard them as *incertae sedis* [16], [17].

**Figure 1:**
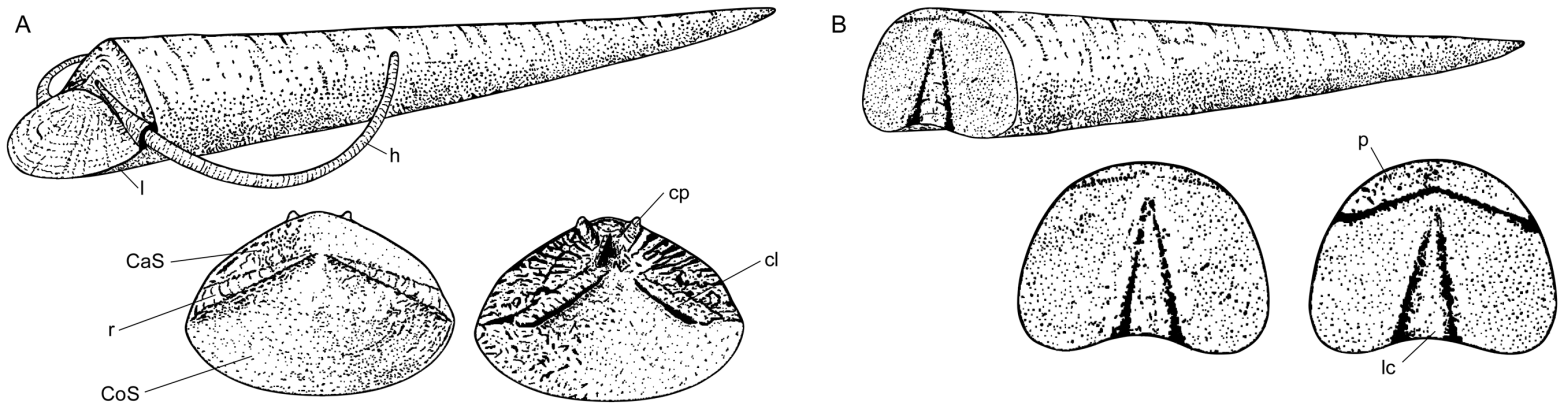
Hyolith terminology. A, Reconstruction of the hyolithid conch *Joachimilites novaki* [23] with proturding helens and *in situ* operculum. External (left) and internal (right) surfaces of operculum are detailed. l: ligula; h: helens; CaS: cardinal shield; r: rooflet; CoS: conical shield; cp: cardinal process; cl: clavicle; modified after [8], [56]. B, Reconstruction of the orthothecid conch *Nephrotheca housina* [23] with *in situ* operculum and detail of external (left) and internal (right) surfaces of operculum. p: process; lc: clavicle-like structure; modified after [23].

Hyoliths are subdivided into two morphologically distinct groups regarded as two subclasses [18] or, as considered herein, orders [5], [6], [10]: Hyolithida [19] and Orthothecida [20]. The hyolithid conch is characterised by a dorso-ventral differentiation based on the presence of a prominent extension of the ventral apertural edge to form a central shelf called the ligula (Figure 1A) [21]. The angular to rounded operculum exactly matches the margin of the aperture (Figure 1A). Lateral slits (rooflets) located between the edge of the operculum and the conch allow a pair of rod-like skeletal elements, called helens, to extend out of the conch (Figure 1A). The helens are long, thin, tapering skeletal elements that, during life, curved ventrally following a logarithmic spiral [22]. The inner surface of the operculum also exhibits well-developed structures, particularly the radially arranged clavicles and the dorsally located paired cardinal processes (Figure 1A) [2], [23]. Orthothecids lack helens and a ligula, but display an even aperture and a planar operculum (Figure 1B) [20]. The internal surface of the operculum is, however, similar to that of the hyolithids due to the occasional presence of processes and clavicle-like folded structures (Figure 1B). The shell of hyoliths, like molluscs, but also the exoskeleton of different marine invertebrates, was originally composed of aragonite [24]. In the hyolithids, the calcium carbonate shell is two-layered. Two microstructures (similar to molluscs) are recognised: prismatic and cross-lamellar layers [11], [25] or two layers of crossed lamellae of fibres [11]. In the orthothecids, two layers of fibrous, tubular bundles are longitudinally arranged in the external layer and transversely structured in the inner layer [15]. Even if the crossed-lamellar structure is otherwise only known in molluscs, this microstructure is derived within the clade [12], and the orthothecid microstructure seems divergent from the mollusc shell-fabric [15]. However, the presence of fibrous calcitic prisms in some brachiopods also suggests that shell fabrics might be convergent among the metazoans [11]. Except in the acuminate or bulbous apical region (the protoconch), the skeletal elements of hyoliths exhibit comarginal growth-lines indicating that these skeletal components were periodically secreted by an epithelium [11]. However, in contrast to this general accretionary growth, some authors [26] presented evidence of an additional inner fibrous layer in the helens of hyolithids which growths without incorporation of previous ontogenetic stages. The hyolith larval development is comparable to that shown by some extant molluscs [7].

**Figure 2:**
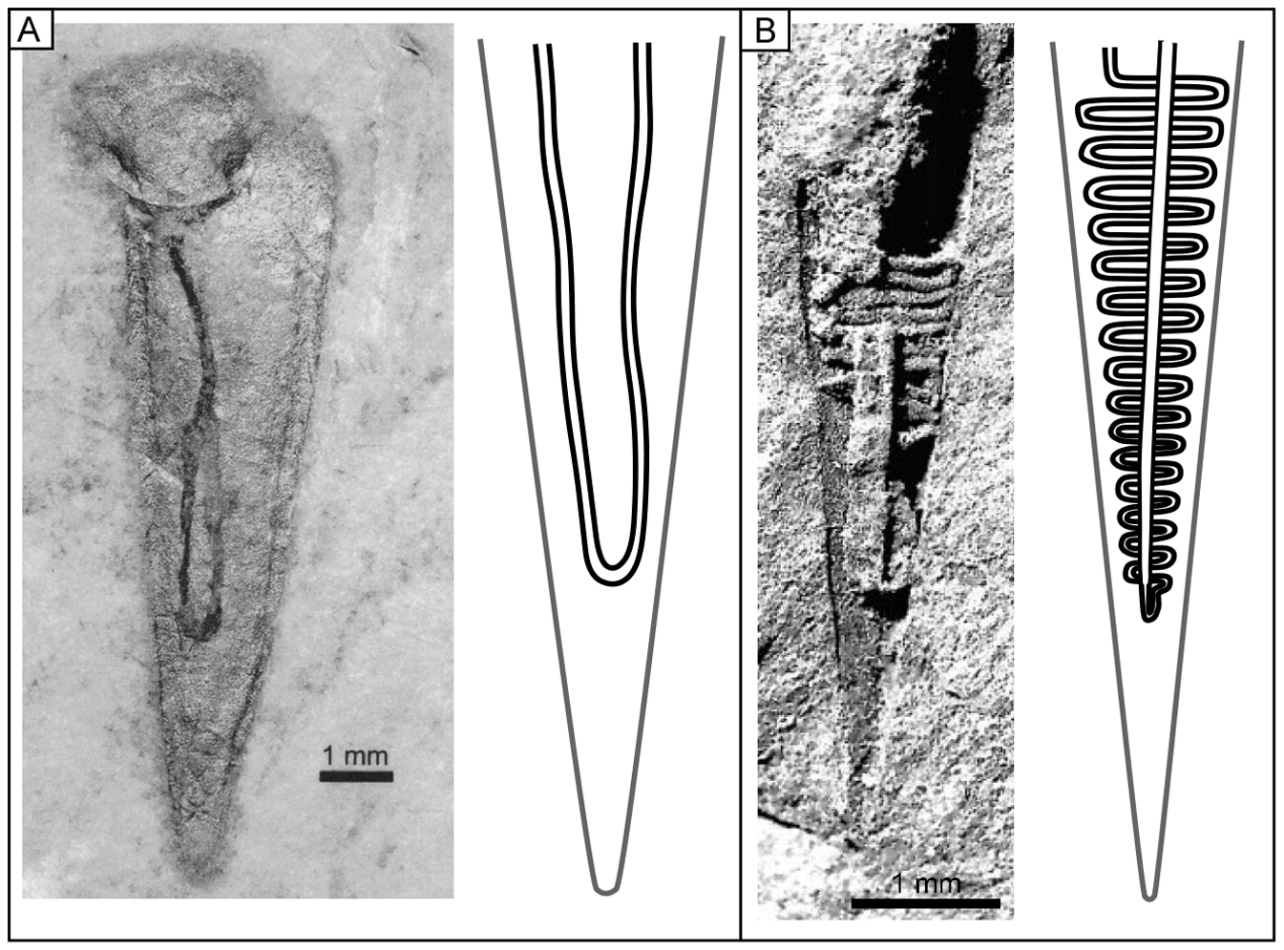
Hyolith digestive tracts. A, Hyolithid specimen with three-dimensionally preserved digestive tract from Cambrian Stage 4 of northern Canada, reprinted from [30] under a CC BY license, with permission from Oxford University Press, original copyright 2003, and reconstruction of the simple U-shaped hyolithid digestive system. B, Specimen of the orthothecid *Girvanolithes thraivensis* [75] with three-dimensionally preserved digestive tract from the upper Ashgill of the Girvan district, Scotland, reprinted from [33] under a CC BY license, with permission from Paleontological Society original copyright 2003, and reconstruction of the partially folded U-shaped orthothecid digestive system.

Differentiation between members of the two orders also relies on their soft parts (Figure 2), although only a few occurrences of preserved hyolith soft parts are reported: in the lower Cambrian of Siberia [27] and possibly China [28]; the middle Cambrian of Antarctica [11], USA [29], Canada [30] and Australia [9]; the Lower Ordovician of France [31] and Bohemia [32]; the Upper Ordovician of Czech Republic [23] and Scotland [33]; and the Lower Devonian of Germany [34]. Digestive tracts content generally gives a third dimension to the preserved digestive tracts, even if their spatial configuration may be flattened by taphonomic processes. The configuration of hyolithid digestive tracts was first investigated through preserved traces of hyolithid intestines in the middle Cambrian Burgess Shale [29] and Kaili formations [35]. The digestive tract appeared not to be sinuously folded although further investigations were precluded by poor preservation. Better preserved hyolithid digestive tract allowed the identification of simple-folded tracts (U-shaped tracts with straight branches; e.g., Figure 2A) [36], [37]. On the other hand, the orthothecid digestive tract was well-documented [9], [11], [23], [27], [31], [32], [33] and consists of a branch of the U-shaped intestine sinuously folded into tight zigzags while the other branch is straight (Figure 2B). The tight zigzags could even be secondarily folded at the midline, forming chevron-like structures (Figure 2D) [9], [27].

This study describes an unusually abundant occurrence of fossilised guts in an orthothecid species from the early Cambrian (Terreneuvian) of southern France, representing one of the oldest records of such a preservation and providing key information on the phylogenetic relationship between hyolithids and orthothecids. The species in question is *Conotheca subcurvata* [38] which occurs abundantly in the Nemakit-Daldynian to Tommotian (Terreneuvian) of northern Montagne Noire (southern France) [39], [40], [41]. This phosphatised orthothecid material is exceptionally preserved in that numerous specimens of internal moulds exhibit three-dimensionally preserved digestive tracts. Preserved intestines were not reported in the very first exhaustive systematic descriptions of the diverse and abundant Heraultia Limestone fauna [39], [40]. Twelve specimens with preserved digestive tracts have been subsequently reported from this limestone and described [42], [43] (see critical discussion in [10], and below). About 285 specimens recovered during the revision of the Heraultia Limestone fauna [41] allow the detailed description of these three-dimensionally preserved digestive tracts. They also provide information about the ontogeny of *C. subcurvata* and about the juxtaposition of its conch and operculum in life.

### Geological setting

**Figure 3:**
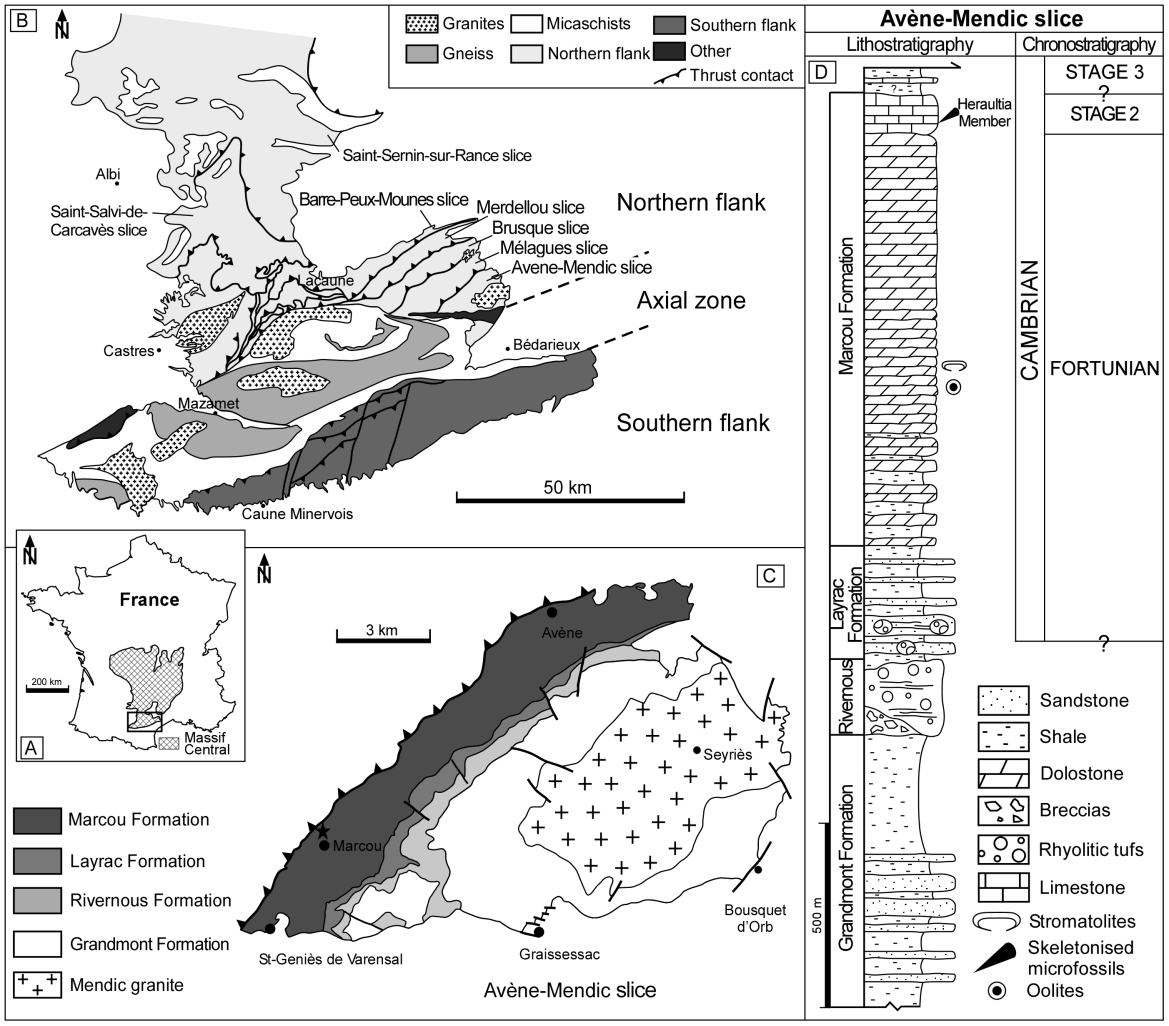
Geological features of the Montagne Noire. A, Location of the Massif Central and the Montagne Noire in France. B, Structural sketch of the Montagne Noire, after [41], [46], [49]. C, Geological sketch of the Avène-Mendic parautochthon with location of the sections yielding hyolith with three-dimensionally preserved digestive tracts, after [41], [48], [49]. D- Litho- and chronostratigraphic setting of the Avène-Mendic parautochthon, after [41].

The Montagne Noire is located south of the Massif Central (Figure 3A). It corresponds to a segment of the southern external zones of the Variscan belt. It is surrounded by post-orogenic sediments from the latest Carboniferous upwards. The Montagne Noire is structurally subdivided into three domains (Figure 3B) [44], [45]: (1) a metamorphic Axial Zone [46], separating (2) the imbricate nappes of the southern flank, in which lower Cambrian to Carboniferous sedimentary rocks outcrop, from (3) the northern flank, involving thrusts slices bearing lower Cambrian to Silurian strata [47]. The studied Heraultia Limestone Member outcrops in the Avène-Mendic parautochthon [39], [40], the easternmost slice of the five recumbent units constituting the eastern termination of the northern flank (the Eastern Lacaune Moutains; Figure 3C). The four other slices are the Mélagues, Brusque, Merdellou and Barre-Peux-Mounes slices.

The lower part of the revised lithostratigraphic scheme of the northern Montagne Noire [48] consists of a thick volcanosedimentary complex, while its upper part is made up of a carbonate dominated sedimentary succession called the Marcou Formation (Figure 3D). This formation represents the onset of carbonate productivity in the platform and has been subdivided into three members (from bottom to top): (1) 200–300 m of dolostone/shale alternations; (2) a massive dolostone accumulation, up to 900 m thick and, (3) the Heraultia Limestone Member, 60 m thick.

The Heraultia Limestone Member was deposited through repeated cycles of carbonate sedimentation, phosphate concentration and reworking which took place from a stable inner shelf to an unstable slope-to-basin sea-floor [49], [50]. Intense microbial activity leads to the precipitation of phosphate crusts indicating the hardground levels, and to the early phosphatisation of the abundant bioclastic content [49], [50].

The reevaluated Terreneuvian age (Nemakit-Daldynian to Tommotian according to the Siberian Chart) of the Heraultia Limestone Member has been determined following a recent exhaustive revision of its abundant and diversified microfauna [41].

## Materials and Methods

Numerous specimens of *Conotheca subcurvata* [38] with three-dimensionally preserved digestive tracts were recovered from the abundant and diversified previously described [39]–[41] phosphatic microfauna from the Heraultia Limestone. The fauna from the Heraultia Limestone has been analysed through sampling of four sections (Figure 3C): K2 and K3 in the vicinity of Marcou village, northwards to Saint Geniès-de-Varensal, and K4 and K5 on the northern and southern banks of the Bouissou river, upstream from Saint Geniès-de-Varensal (see [41] for sections and sampled horizons). The studied phosphatised specimens were obtained by ca. 10% acetic acid treatment of the limestone samples. They were picked from residues under a stereo-microscope, and then studied with a Zeiss Supra 40 VP Scanning Electron Microscope at the Freie Universität Berlin and a FEI Quanta 200 at the University of Lille 1. Thousands of *C. subcurvata* specimens, homogeneously distributed through the sections, were recovered (see figs. 29–33 in [41] for detailed stratigraphic distribution); only 285 specimens (less than 10 percent) have partial to complete preservation of the gut. They were recovered from samples K2/4,6,8,13; K3B/13; K3T/2–4; K4/4,9,17,21; K5b/8; K5/16–17,19,35,39–40 (see [41]). Although they are more numerous in beds K2/4 (confidently identified as previously studied by [42]), K3T/2 and K4/4, their relative abundance in the various samples is not significantly different from the total richness of the recovered microfauna. This observation differs from the conclusion of a previous study [42] in which preservation of guts was interpreted as unexplained and preferential, in just a few horizons of the therein studied section.

About 10 specimens have been sectioned in order to analyse the structure and composition of the content of phosphatised gut through SEM (Back Scattered Electron) and Energy Dispersive Spectroscopy. Measurements were made from digital SEM photographs using ImageJ [51] and statistics run with PAST [52]. Figured specimens are housed in the palaeontologic collection of the University Lille 1, France (acronym USTL). No permits were required for the described study, which complied with all relevant regulations. The material used in the present study was extracted from the public domain in a non-protected area, an unregulated practice in France. The present material is deposited in public collections and is the property of the French Republic and has not been the subject of any mercantile activity.

## Results

### Systematic Palaeontology

Phylum uncertain. *Remarks.* To date, hyoliths share most similarities with the molluscs with which they may be related. However, recent phylogenetic schemes based on palaeontologic and molecular data have not demonstrated any relationship between molluscs and hyoliths. Such a discussion is out of the scope of this paper and the systematic position of hyoliths is herein considered as uncertain at phylum level.

Class HYOLITHA Marek, 1963 [2]

Order ORTHOTHECIDA Marek, 1966 [20]

Family CIRCOTHECIDAE Missarzhevsky, 1969 in [4]

Genus CONOTHECA Missarzhevsky, 1969 in [4]

*Type species. Conotheca mammilata* Missarzhevsky, 1969 in [4]; lower Cambrian, Tommotian (*Dokidocyathus lenaicus-Marjatheca tumefacta* Zone), middle course of the Lena River, Churan village, Siberian Platform, Russian Federation.

*Conotheca subcurvata* Yu, 1974 [38] emend.

**Figure 4:**
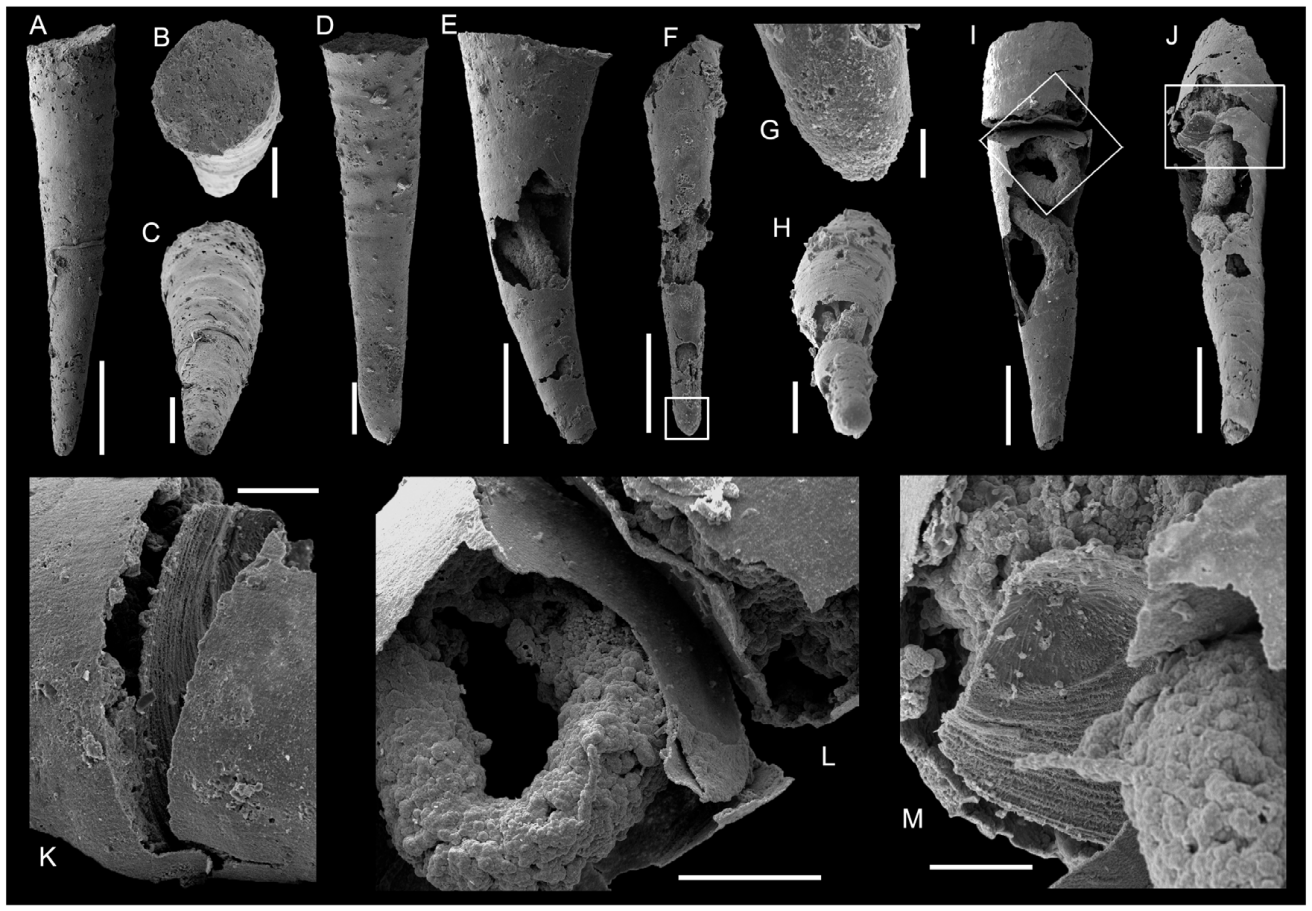
*Conotheca subcurvata* [38]. A-C, USTL1264/3; phosphatic internal mould; A- Lateral view; B, Oblique view of the circular aperture; C, Oblique view of the blunt apex. D, USTL1210/9, Lateral view of internal mould. E, USTL2786/5, lateral view of internal coating of conch with phosphatised guts visible. F-H, USTL2785/5; F, Lateral view of internal coating of conch with phosphatised guts visible, squared area magnified in G; G, Detail of blunt apex; H, Oblique apical view of blunt apex. I-M, USTL2786/1; I, Lateral view of internal coating of conch with coating of operculum and preserved guts, squared area magnified in L; J, Oblique lateral view, showing the lateral continuity of conch coating (right-hand side), squared area magnified in M; K, Detail of junction of operculum and conch coatings; L, Detail of operculum coating and folded digestive tract; M, Detail of coating-imprint of operculum external surface. Scale bars are: G, K, 100 µm; B, C, H, L, M, 200 µm; A, D, E, F, I, J, 500 µm.

**Figure 5:**
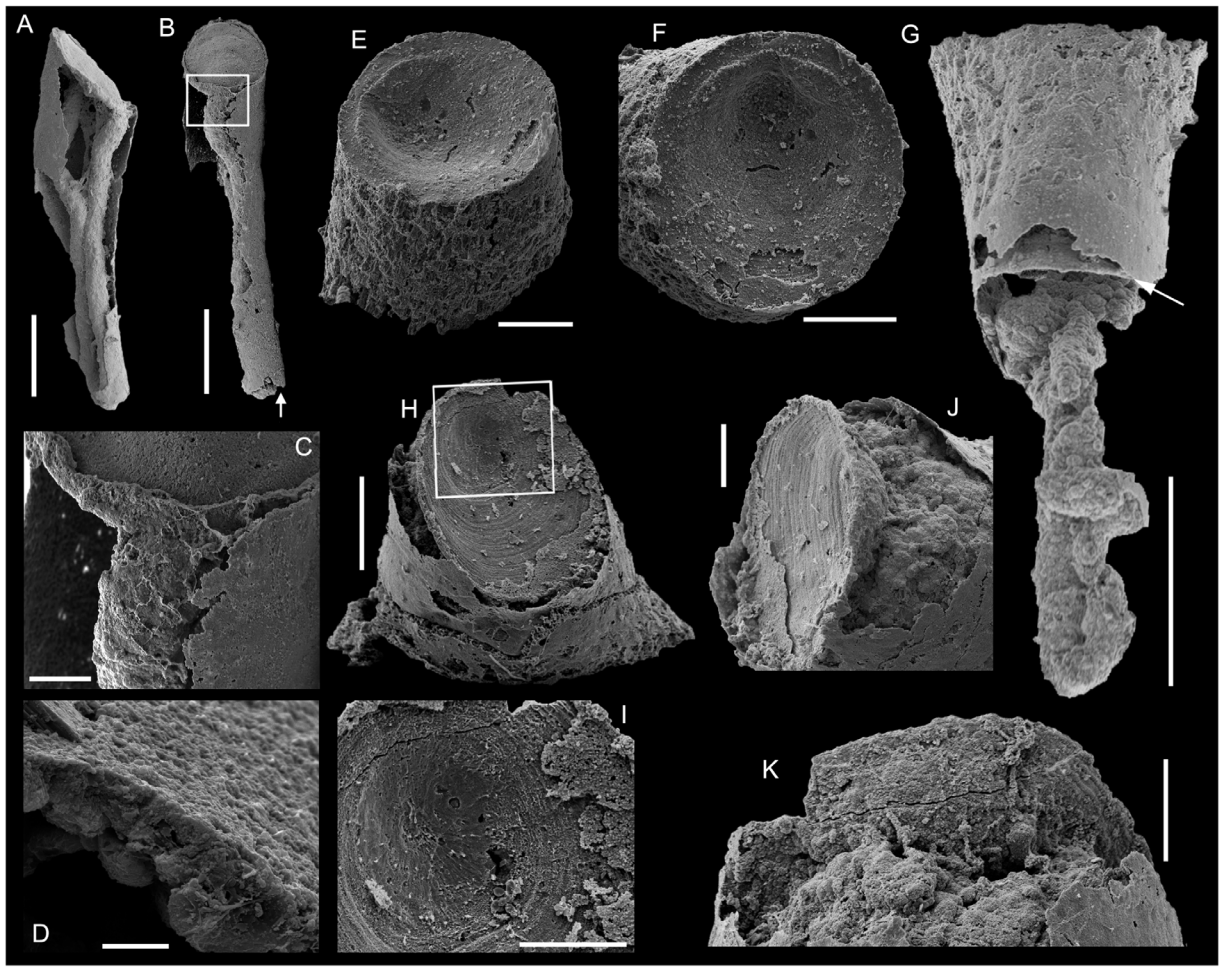
*Conotheca subcurvata* [38]. A-C, USTL1251/13; A, Lateral view of broken internal coating of conch with preserved digestive tract; B, Oblique apertural view with internal surface of phosphatic coating of operculum visible, squared area magnified in C and orientation of D arrowed; C, Detail of proximal round extremity of the phosphatised gut; D, Inner coating of the conical conch by lining biofilms made of amalgamated coccoids. E-F, USTL2785/3, apertural phosphatic exterior compartment with coating of operculum inner surface; E, Oblique lateral view; F, Internal view. G, USTL2783/4, lateral view with detail of junction of operculum and conch coatings. H-K, USTL2782/13; H, Coating of operculum external surface, squared area magnified in I; I, Detail of radially ornamented embryonic part of the operculum coating; J, Oblique lateral view of operculum external surface coating and proximal compartment with coccoidal phosphate filling; K, Lateral view of transition between withdrawn operculum and proximal compartment with coccoidal phosphate filling, filaments on operculum external surface. Scale bars are: D, 10 µm; C, I-K, 100 µm; E, F, H, 200 µm; A, B, G, 500 µm.

**Figure 6:**
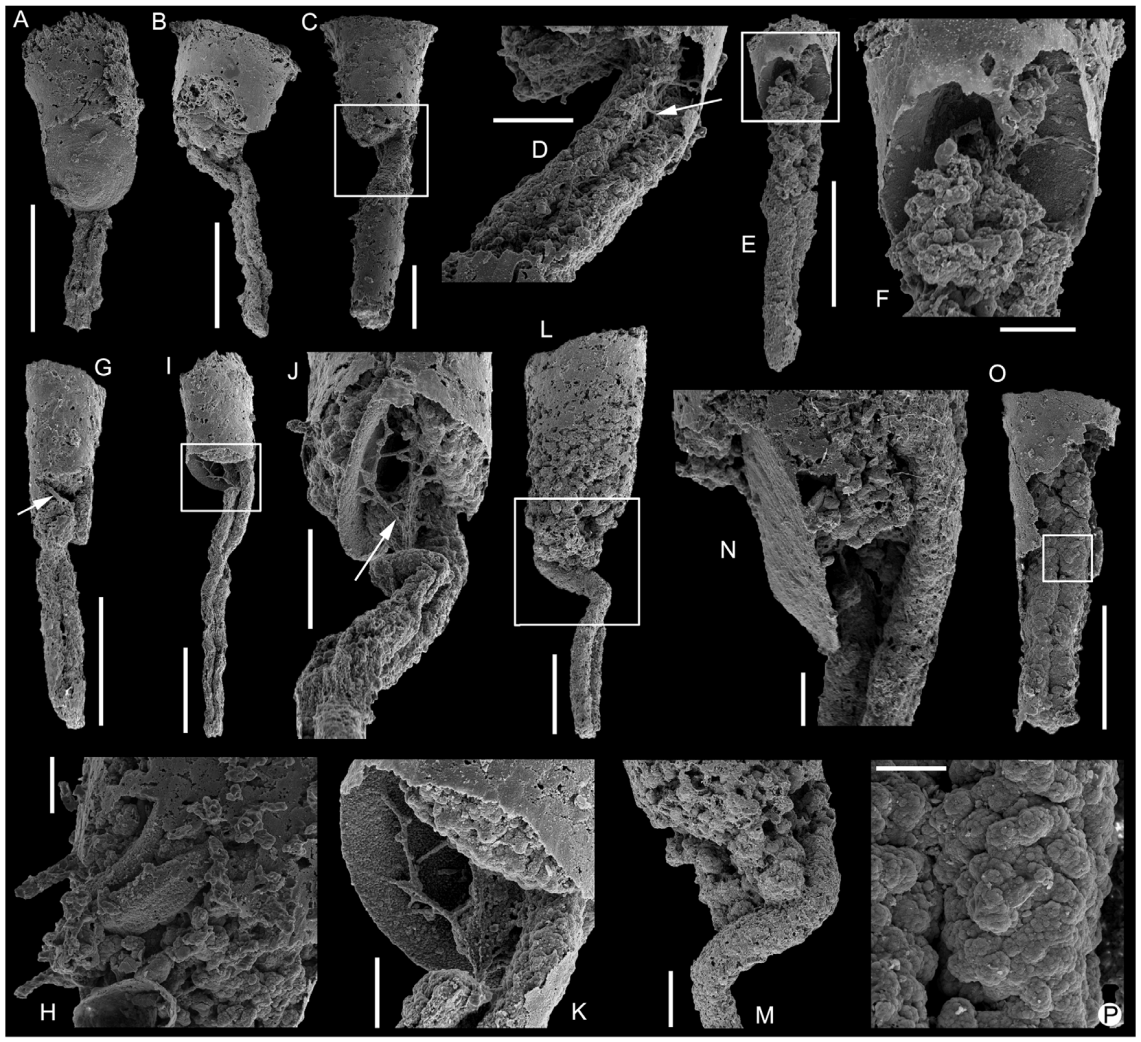
*Conotheca subcurvata* [38]. A, USTL2782/11; Lateral view showing incompletely preserved guts and external coating of withdrawn operculum. B, USTL2782/3; Lateral view of specimen with different microbial pseudomorphs in the internal and external compartments and overgrowth. C-D, USTL2782/2; C, Lateral view, squared area magnified in D; D, Detail of the preservation of the digestive tract and filamentary junction with mould (arrow), the filaments seem to cover or invade the pre-existing mould. E-F, USTL2788/7; E, Lateral view, squared area magnified in F; F. Detail of Large coccoids covering the intestine cast and preserved withdrawn operculum. G, USTL2788/8; Lateral view of conch, exogenous material (tube) in the exterior compartment arrowed. H, USTL2784/2; Detail of exogenous material (halkierid) within the conch. I-K, USTL2781/6; I, Lateral view, squared area magnified in K; J, Possible fungal hyphae (arrowed) in connection with intestine, in this case communication with exterior compartment; K, Detail of possible hyphae. L-M, USTL2789/2; L, Lateral view, squared area magnified in M; M, Intestine phosphatic meshwork denser than exterior compartment. N, USTL2789/3; Detail of relations between exterior and interior compartments and operculum. O-P, USTL2781/9; O, Lateral view, squared area magnified in P; P, Detail of diagenetic overgrowth of the intestine phosphatic cast. Scale bars are: P, 50 µm; F, H, K, N, 100 µm; D, J, M, 200 µm; A-C, E, G, I, L, O, 500 µm.

**Figure 7:**
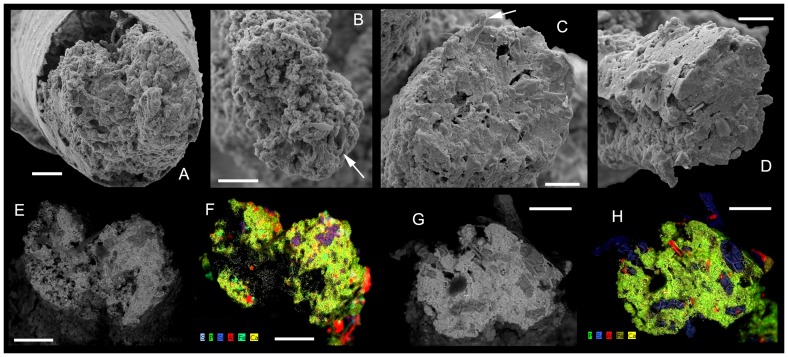
*Conotheca subcurvata* [38]. A, USTL2784/1; Detail of distal section of digestive tract, gut content phosphatisation shows typical microbial mediation with filamentary pseudomorphs. B, USTL2784/5; Detail of section of digestive tract, gut content phosphatisation shows typical microbial mediation with filamentary pseudomorphs. Exogenous material (biogenic tube) in gut content arrowed. C, USTL2784/2; Detail of section of digestive tract with overgrown quartz inserted in the structure (arrowed). D, USTL2784/7; Detail of section of digestive tract showing quartz inserted within the structure. E-F, USTL2784/6; E, BSE image of section of digestive tract; F, Corresponding EDS analysis showing the occlusion of the porous gut by terrigenous material (mainly quartz and mica) cemented by apatite and calcite. G-H, USTL2784/7; G, BSE image of section of digestive tract; H, Corresponding EDS analysis showing terrigenous material as intraparticular infill subsequently cemented by apatite. Scale bars are: B-D, 50 µm; G, H, 60 µm; E, F, 80 µm; A, 100 µm. Al  =  chlorite, P  =  apatite, Ca  =  calcite, Si  =  quartz, Fe  =  pyrite.

**Figure 8:**
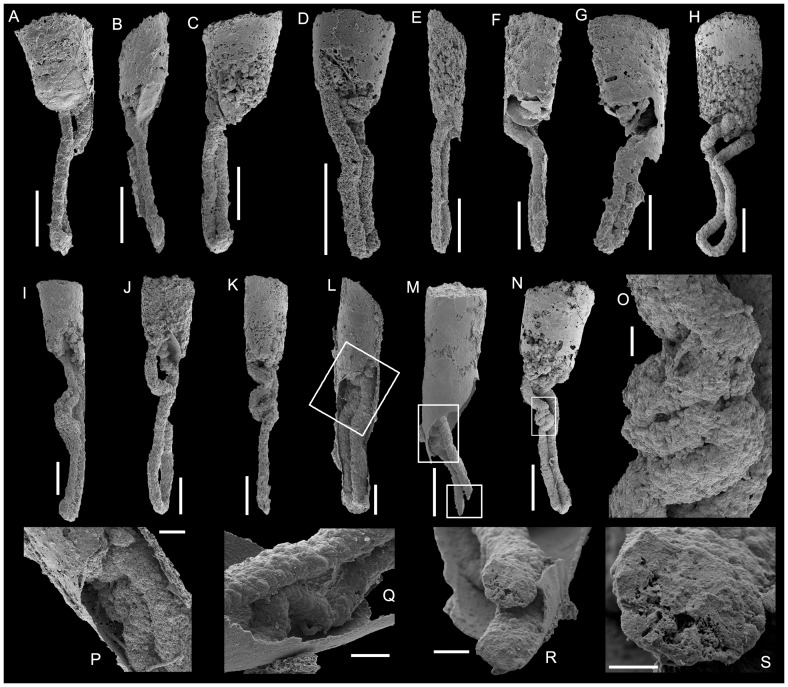
*Conotheca subcurvata* [38]. A-B, USTL2781/5; A, Lateral view of the conch with external surface of withdrawn operculum visible; B, Gut protruding out from a narrow gap between the withdrawn operculum and the conch internal coating. C-H, Specimens with intestine type 1 (gut and anal tube of the U-shape intestine straight or slightly undulating and parallel to each other); C, USTL2782/6; D, USTL2781/8; E, USTL2787/10; F-G, USTL2783/9; H, USTL2791. I-J, Specimens with intestine type 2 (straight anal tube and a portion of the gut loosely folded); I, USTL2785/1; J, USTL2787/11. K-S, Specimens with intestine type 3 (straight anal tube and a portion of the gut tightly folded into zigzags); K, USTL2786/2; L, USTL2783/2, squared area magnified in P; M, USTL2789/1, upper squared area magnified in Q and lower squared area magnified in R, N, USTL2790, squared area magnified in O; O-Q, Tightly folded portion of the gut; R, Detail of a cross-section of the gut; S, Detail of the upper cross-section in Q. Scale bars are: O, S, 50 µm; Q, R, 100 µm; C, P, 200 µm; A-B, D-N, 500 µm.

Figures 4-8. *Synonymy list.* See [41].

*Emended diagnosis.* Narrow conical straight to slightly curved conch, circular to oval in cross-section. Apertural margin straight and apex blunt. Internal conch surface smooth, external surface with faint and dense growth lines. External surface of low conical operculum with an eccentric, smooth and rounded summit and concentric growth lines. Internal surface of the operculum with offset marginal-zone only.

*Remarks.* The association of an operculum and almost complete conch has been found in at least two specimens (Figures 4I-M, 5A-C), while 25 additional specimens preserve the operculum with part of the conch. Therefore, the diagnosis of *Conotheca subcurvata* is completed herein in order to take into account the newly described operculum. The internal structure of the operculum is rather simple and fits the diagnosis of Orthothecida, supporting the assignment of *Conotheca* to that order.

*Material.* Thousands of phosphatic internal moulds and hundreds of internal coatings. 285 specimens preserved as phosphatic internal moulds of the conch in the apertural region and as phosphatic moulds of the digestive tracts in the apical part, including illustrated specimens USTL 1210/9, 1251/13, 1264/3, 2781/5–6, 2781/8, 2781/9, 2782/3, 2782/6, 2782/11, 2782/13, 2783/1–4, 2783/9, 2784/1–2, 2784/5–7, 2785/1, 2785/3, 2785/5, 2786/1–2, 2786/6, 2787/10–11, 2788/7–8, 2789/1–3, 2790, 2791.

*Description.* Complete conchs are preserved as internal moulds. The narrow conical conchs are straight (Figures 4A, 4D, 4F, 4I) to slightly curved (Figure 4E), circular (Figure 4B) to slightly oval (Figure 5B) in cross-section. The angle of divergence is uniform through the entire length and ranges from 8 to 15°, the length between 1 to 4 mm and the diameter from 0.33 to 1.2 mm. The apertural margin is circular (Figure 4B) to slightly oval (Figure 5B). It is straight in lateral view and perpendicular to the longitudinal axis (Figures 4A, 4D, 4E, 4I). The apical extremity is blunt (Figures 4A, 4C, 4D, 4F, 4G). The external surface of the internal moulds is smooth, with low ribs or undulations (Figure 4D).

The concavo-convex operculum is rarely preserved but usually located more or less deeply into the conch (Figures 4I-J, 5E-K, 6A, 6E, 6I-K, 6N, 8A-C, 8F-G vs 5A-C). It may be preserved parallel to the apertural plane (Figure 4I) or inclined with an angle up to 50° from it (Figures 6A, 6E, 6I-K, 6N, 8A-C, 8F-G). It is preserved as a phosphatic coating of the original calcium carbonate material dissolved during acetic acid treatment (Figures 4I-N). It is circular to slightly oval in outline (Figures 5B, 5E, 5F, 5H) with a faint eccentric summit (Figures 5B, 5E, 5F, 5H) that is located about 1/4 of the distance from the dorsal(?) border to the ventral(?) margin (Figures 5B, 5E, 5F, 5H) and rounded (about 150 µm in diameter). The external surface of the operculum is convex and ornamented with comarginal growth lines (Figures 4K-M, 5H-J) except for the smooth to radially ornamented summit area (Figures 4K, 4N, 5I). In lateral view, the operculum is low conical (Figures 5E, 5G, 5J) but sometimes almost flat (Figures 5A, 4I, 4L, 8B). The internal surface of the operculum is concave and exhibits a distinct offset marginal zone with a width of about 1/10 of the diameter (Figures 5E-F). The maximum thickness of the operculum is achieved at the inner margin of the peripheral offset. Processes and clavicle-like structures are absent.

*Comparison.* Comparisons with other species of *Conotheca* are difficult because opercula are rarely preserved attached. The latter are only described in *C. australiensis* [16], *C. rotunda* [53] and *C. brevica* [54]. In these three species, the operculum exhibits an offset marginal zone similar to that of *C. subcurvata*. However, opercula of *C. australiensis, C. brevica* and *C. rotunda* have paired processes and *C. australiensis* and *C. rotunda* have clavicle-like structures that are all absent in *C. subcurvata*. Few characters of the conch itself are diagnostic (mostly related to cross-section, aperture, apex and angle of divergence). The angle of divergence in *C. subcurvata* is uniform, which differs from *C. mammilata* [4]. The species *C. circumflexa* [4] is characterised by a slightly helical growth component of the conch, not present in *C. subcurvata*. On the other hand, *C. shennongjiaensis* [55] has a very sharp apex when compared to *C. subcurvata*. However, given that *Conotheca* species have a very simple shell and the various records of opercula are quite different (opercula with or without processes within the same genera), the species of *Conotheca* may not be closely related. Thus, the different species listed under the genus *Conotheca* may correspond to separate genera. Since the operculum is not known in the type species *C. mammilata*, its precise relationship to other species referred to the genus cannot be ascertained. Both the operculum and conch should be used for the definition of any hyolith genus [56], [57] although the absence of opercula associated with conchs is recurrent due to sorting, post-mortem destruction, and possibly to a different composition from the conch material [56].

An earlier study of twelve specimens of orthothecids from the Heraultia Limestone with preserved gut and three additional specimens showing the presence of poorly preserved and/or fragmented operculum informally referred the gut-bearing specimens to three different genera (possibly five) without any assignment at species level or formal systematic description ([42], see also comments in [10]). The present description of abundant and better preserved specimens (some notably showing gut and shell characters in association) as part of an exhaustive study of the Heraultia Limestone fauna [41] allows the confident reinterpretation of those specimens as belonging to *C. subcurvata*.

### Taphonomic and taxonomic trends of phosphatised digestive tracts

The Heraultia Limestone consists of bioclastic limestone strata rich in microbially mediated phosphorites, from which the studied orthothecids were extracted. The biogenicity of the structures and microbial activity is based on several lines of evidence, including comparison of the abundant phosphatised pseudomorphs with biogenic morphology and behaviour reported in modern mat systems with rapid mineralisation [50]. Four different microbial pseudomorphs are identified in the studied phosphorites: (i) smooth uniseriate to dichotomously branched filaments (2–50 µm in diameter); (ii) thin, segmented filaments (4–6 µm in diameter) otherwise similar to the first type; (iii) filamentous structures with circular to multilobate cross-section (100–150 µm in diameter) made of amalgamated ovoid units; and (iv) botryoidal assemblages of coccoid morphotypes (50 µm in diameter). Detailed diagenesis and microbial ecology of the phosphorites have been extensively described [49], [50]. We will focus below on the microbial consortia associated with *C. subcurvata* and, in particular, its previously undescribed relations to digestive tract and operculum.

Specimens of *C. subcurvata* show a typical preservation as inner phosphatic, microbial and/or sediment internal mould (Figures 4A-D), or inner coating of the conical conch by lining biofilms (Figures 4E, 4I-J, 5A-D) made of amalgamated endolithic, coccoidal and filamentary microbes (Figure 5D). These biofilms are relatively thin, up to 10 µm thick. In conch-operculum associations, the operculum is possibly located up to about 250 µm into the conch although it is situated at the preserved conch margin in one specimen (USTL1251/13; Figures 5A-C). The opercula are also preserved as phosphatic, endolithic microbial coatings which developed in continuity with the conch inner-cast and partially replicate its original ultrastructure (Figures 4I-L, arrowed in 5G). Where preserved transverse or in a slightly oblique position inside the conch, the opercula perfectly fit the conch diameter so that they subdivide the conch into two compartments (e.g., Figures 4I, 4J, 5G, 6E): for convenience, the compartment which opens at the conch margin is here called exterior, while the operculum in its preservational position delimits an inner compartment. These two compartments can be independently preserved (Figures 5E-F, 5H-K, 6O) or associated by the fragile operculum peripheral-coating which is fused to the shell coating (Figures 4I-J, 5G, 6E-F, 6I-K). The exterior compartment is usually filled with secondarily phosphatised sediment and debris (tube arrowed in Figure 6G and halkieriid sclerite in Figure 6H) and/or by a network of loose to dense botryoidal amalgamation of hollow to infilled, coccoidal pseudomorphs (about 50 µm in diameter; Figures 4M, 5J-K, 6B, 6J, 6N, 8C) associated with thin filaments (5–10 µm in diameter; Figures 5J-K, 6J). The inner compartment is generally exempt from microbial invasion and preserved empty of phosphatisation, except for the preserved gut. Where the operculum was absent or strongly oblique, the inner compartment directly opened to the exterior has subsequently been invaded by a microbial consortium (Figures 6C-D, 6I-J, 6N). Two of the different described pseudomorphs are identified in relation with the hyolith intestines: smooth uniseriate, branching filaments (Figures 6C-D, 7A) and phosphatic coccoids (Figures 4E, 4L, 5G, 8O, 8Q) act in various proportions in the dense network which apparently casts the digestive tract. This network is generally denser than the botryoidal structures and filaments reported from the exterior compartment (Figures 6B, 6L-N). The phosphatised coccoid morphotypes can be preserved as isolated structures on the outer surface of the infilled guts, although they currently form superposed coats which have been slightly and variably overgrown (irregular surface texture; Figures 6O, 6P). This preservation bias precludes any interpretation of the variation in gut diameter as an ontogenetic pattern, even if overgrowth seems to be generally low.

The sections of digestive tracts analysed through SEM (Back Scattered Electron) and Energy Dispersive Spectroscopy show a porous structure lacking authigenic, massive phosphate crystallisation. In some specimens, gut phosphatisation results from typical microbial mediation with small coccoidal (10 to 20 µm in diameter) and filamentary (c. 10 µm in diameter) pseudomorphs (Figures 7A, 7B). Even if some subordinate terrigenous material might represent pollution during sample preparation, quartz, mica and argillaceous material are clearly embedded in the fabric (Figures 7E-G), subsequently cemented with apatite and calcite, and even diagenetically overgrown leading to automorph outlines (Figures 7D, 7G-H and arrowed in Figure 7C). These features point to their role as original infill (Figures 7C-D). Fragments of biogenic tubes (hyolithelminth-like tubes, arrowed in Figure 7B) are also part of the gut infill. The presence of non-phosphatic remains, such as quartz, mica or argillaceous material, suggests the input of sediment into the gut prior to apatite precipitation and calcite occlusion of the remaining porosity. Incorporated bioclasts further argue for a deposit-feeding strategy of the orthothecids. The internal structure of the gut and the presence of gut content were not recognised in previous studies [42], neither were the spatial relationships between the preserved-gut, operculum (no direct contact between the structures were observed, fig. 6.7 in [42]), and the phosphatic-plug (called exterior compartment herein). Moreover, the rapid overview of involved phosphatisation processes lead to the interpretation of a twofold phosphatisation process (gut and shell coating and then ‘plug’ phosphatisation, p. 189 in [42]). The present detailed description allows the identification of the more complex taphonomic history.

As already discussed in [49], [50], the phosphate accumulation occurred in a complex sedimentological context of repeated cycles of hardground condensation, skeletal phosphatisation and reworking. The skeletal remains were the loci of stepwise invasion by microbial consortia. The new specimens of colonised conchs of *C. subcurvata* delimit the colonisation pattern with some precision. The inner surface of the shells, in both external and internal compartments, is currently coated by microbial films (Figures 4E, 4I, 5A-D). The opercula, where present, are also coated on their outer and inner surfaces (Figures 4I-M). However, preservation of the operculum in transverse position limits further microbial development to the exterior compartment and precludes any significant colonisation of the internal compartment (Figures 4I-J, 5G). This colonisation only occurs when direct communication with the exterior is allowed by the loss or displacement of the operculum during decay and reworking (Figures 6E-F, 6I-K). Two independent phases of colonisation are evident: (i) most probably euendolithic and cryptoendolithic consortia restricted to the shell substratum, and (ii) a chasmolithic consortium which invaded the shell cavity from its natural aperture. In our study, possible fungal hyphae were only identified in the chasmolithic association; otherwise, except from botryoidal assemblages of large empty coccoids which are also restricted to the external compartments, no further identification of group(s) involved in the two strategies can be inferred. Moreover, the irregular migration front within the shell provides evidence of secondary covering of the preserved gut by the chasmolithic association.

Described taphonomic changes appear to involve tissue-dependant phosphatisation during decay as only the guts were preserved in *C. subcurvata*. Preserved muscular tissues, which are otherwise greatly involved in the debate regarding the phylogenetic affinity of the group [3], [11], are absent. Only the gut content (and not the guts themselves) has been phosphatised with possible overgrowth so that there is no actual soft-tissue mineralisation in the observed specimens. The gut is apparently preserved independently of the occlusion of the internal compartments by the operculum and of any contact with the shell coating. This suggests the digestive tract represented a third ecological niche for cryptic microbes, the colonisation and phosphatisation of which started early, prior to the decay of muscles and other tissues which supported the gut [37], [58], and prior to any other microbial colonisation of the shell. This is supported both by the three-dimensionally preserved configuration of the gut fill and by the spatial relationship to other preserved elements.

The fact that the three-dimensionally preserved configuration of the digestive tract of *C. subcurvata* was not or only slightly affected by gravitational deformation due to decay of supporting tissues argues for a rapid phosphatisation in a restricted environment. It further suggests the gut itself might has been the source of phosphorus during decay and was initially maintained by ingested material. This, along with the terrigenous content of the intestine, supports the general interpretation of orthothecids as deposit-feeders [6], [9], [11], [14], [29]. Thus, organic-rich ingested sediments might have acted as relative stiffener which helped to prevent gravitational and other deformations during decay and acted as a source of phosphorus for autolithic mineralisation of colonising microbial consortia [59]. As recently demonstrated [60], preserved guts of naraoids (arthropods) from Burgess-Shale type deposits were initially filled by organic rich material and were primarily preserved by early authigenic mineralisation in association with decay prior to further divergent diagenesis (formation of carbonaceous material, calcium phosphate and iron sulfide) and weathering (oxidation, loss of sulphur and calcium and alteration of pyrite to limonite). Accumulated pellets in the Alum Shale Formation from Sweden might have acted as a phosphorus source for the Orsten-type preservation [61] and phosphorus-rich pellets excreted by modern crustaceans [62].

It is also interesting to note that even if the Heraultia Limestone provides a relatively diverse and abundant fossil assemblage, with about 30 taxa (up to 23 in beds from which preserved guts were recovered) [41], only *C. subcurvata* exhibits preservation of some soft parts, suggesting a taxonomic selection in the observed phosphatisation [37]. Other shelly remains, which might have been potential candidates for soft-part preservation, mostly include helcionellids and other molluscs (e. g., *Watsonella*). It is suggested that the relatively narrow and long conical shell of the orthothecids facilitated a rapid obstruction either by the associated operculum or by proximal sedimentary infill which enabled the soft part to act as a source of phosphorus within a confined environment. In taxa lacking an operculum and with a large opening, the shell is completely filled by sediment (and preserved as internal moulds, see [41]). This latter scenario implies that both the surrounding sediment and organic material acted as a source of phosphate in the Heraultia substrate.

### Spatial configuration of digestive tracts

Some digestive tracts are clearly preserved within the partially broken, inner-coating of the conch, thus allowing the identification of their relationships (Figures 4E, 4I-J, 5A, 8L, 8M). Most often, only the phosphatic internal mould of the external compartment is preserved in association with the digestive tract (Figures 6A-N, 8A-K, 8N). In general shape, the preserved digestive tract corresponds to a simple U-shaped tube with an ovoid (almost circular) cross-section. However, this configuration is controlled by the size of the hyoliths and the position of the operculum. As previously described, the preserved opercula are slightly smaller (ca. 80 to 90%) than the conch apertural diameter. Generally, each operculum is displaced or withdrawn into the conch. In such cases, the two branches of the U-shaped digestive tract (composed of the gut and the anal tube [33]) are parallel, loosely folded, and the apertural extremity of the gut seems to lie parallel to the operculum surface (Figure 4L).

In some cases, the operculum is preserved in a strongly oblique position and delimits a lateral narrow gap, from which the straight gut protrudes without any suggestion of contortion (Figure 8B). These taphonomic artefacts suggest that the gut was neither directly nor indirectly attached to the operculum. The latter might have been retractable through muscle contraction (as suggested by the described muscle insertions [6], [11]) while the gut was pushed and smoothly folded inward as a mechanical repercussion of the contraction of the body cavity. However, the absence of an operculum also favoured the onset of a corridor for microbial invasion of the internal compartment which covers the proximal extremities of the guts. When the internal compartment is preserved from the microbial colonisation, it is possible to observe the faintly rounded extremities of the phosphatised gut (Figure 5C), which also infer the predictably small gap between the operculum and the intestine. The gut is not preserved in contact with the shell coating at least in its proximal part (but see e.g., Figures 8L, 8P) and, in most specimens, was not affected by clear gravitational deformations. In some specimens, the two parallel branches of the gut are angled (Figures 6B, 6I, 6L-M, 8E) and, when the shell coating is preserved, they seem to have joined and rested on it (Figures 8F-G, 8L, 8P). The gut occupies most of the shell cavity in its apical portion and can reach the apex (Figure 5A).

Two other configurations of the digestive tract can be recognised in addition to the pattern with straight or undulating but parallel branches (type 1; Figures 8A-H). They are characterised by a straight branch of the gut (called anal tube [33]) and differentiated by the degree of folding of the other branch (gut *sensu stricto* [33]); in type 2, it is loosely folded (Figures 8I, 8J) and, in type 3, it is tightly folded into zigzags (Figures 8K-Q). In types 2 and 3, the straight and unfolded anal tube demonstrates it was not deformed by operculum retraction or taphonomic bias. Only the simplest U-shaped configuration (type 1 herein) was described previously [42], while the putative loosely folded gut (fig. 6.7.k in [42]) is better seen as a taphonomic artefact quite different from the type 3, orthothecid-like configuration, described herein.

## Discussion

The possibility that the operculum of orthothecids could be retracted inside the conch was previously suggested [3], [20], [63], [42]. Indeed, the authors noticed that the diameter of orthothecid opercula is generally slightly smaller than the diameter of the conch aperture. This observation is confirmed by the present specimens and further supported by the recurrent presence of opercula preserved withdrawn inside the conch. The withdrawn opercula do not correspond to disarticulated opercula mechanically inserted into the conch by currents: inserted shell materials and disarticulated opercula are mostly absent from the assemblage and not observed in thin sections. Moreover, the bioclastic elements are not preferentially oriented in thin sections. The capacity of orthothecids to retract the operculum inside the conch is comparable to several living gastropods using it to seal the soft tissues. The operculum could be considered as a defensive structure as in living gastropods. Withdrawal of operculum prevents predators from reaching the organs by just a simple shallow breakage in the apertural margin. A large amount of shell must be peeled back from the shell margin before bypassing the operculum and getting at the soft parts. The operculum could also be retracted within the conch tube but not enough to seal it. It could therefore facilitate respiration but still prevent predation. Although such a function for the operculum would correspond to a filter-feeding strategy that is not compatible with the presence of exogenous material within the guts.

**Figure 9:**
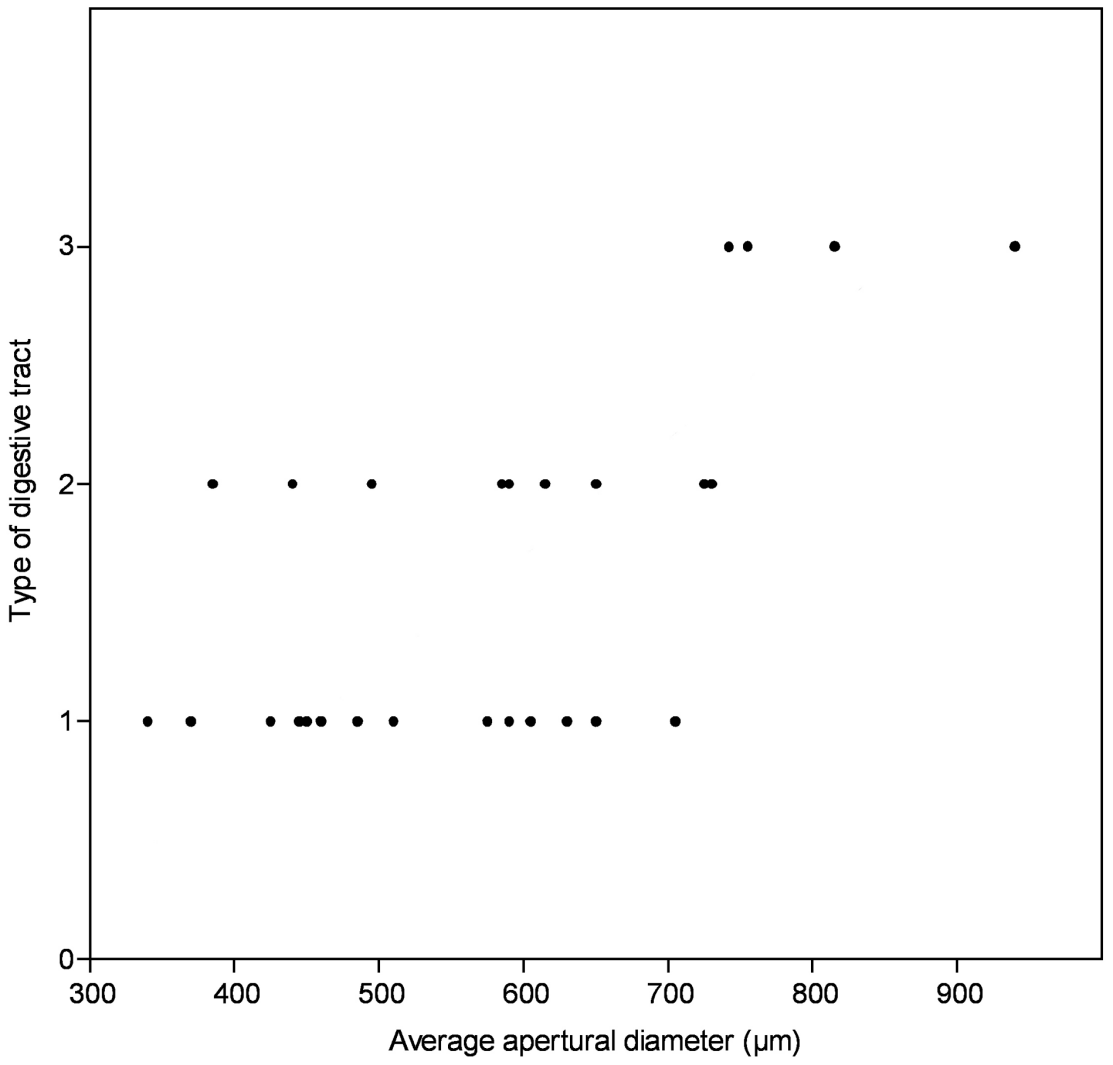
Digestive tract type versus average apertural diameter (µm) based on 27 specimens of *C. subcurvata*. Correlation coefficient: r  =  0,62, coefficient of determination: r^2^  =  0,38 and *p*-value  =  0,00057.

In order to test the ontogenetic significance of the non-taphonomic folding of one branch of the intestine, the three different types of gut configurations were plotted versus the average diameter of the conch at the aperture (Figure 9). To express the growth of *C. subcurvata*, the mean diameter of the conch was measured. The length of most of the conchs could not be estimated as only the apertural phosphatic exterior compartment and guts were preserved. The linear gut length depends on the number of folds and thus it depends on the type of gut configuration. The diameter of the digestive tract is generally taphonomically biased by overgrowth of phosphates. Therefore, only the diameter of the conch can be used to estimate the growth stage. As the conchs are usually deformed (ellipsoidal cross-sections are due to lateral flattening), their average diameter was calculated (mean of maximum and minimum diameter of the ellipse). Among the 285 specimens with preserved digestive tracts, only 27 were sufficiently well-preserved to enable the confident identification of their type of digestive tract and therefore to be included in the data set. The relation between diameter and digestive tract type was statistically tested through linear regression with PAST [52]. The correlation coefficient (r) value is 0.62, meaning that the two variables are linearly correlated at 62%. On the other hand, the value of the square correlation coefficient (r^2^ = 0.38413) indicates that only 38 % of the variance is explained by the linear association of the digestive tract type and the average diameter. However, the positive correlation between the apertural average diameter of the conch and the type of digestive tract is statistically supported (significant at p = 0.0057). Type 3 (straight anal tube and a tightly, zigzag folded portion of the gut) appeared in the largest specimens with preserved intestines. A sinuously-folded digestive tract is characteristic of the Orthothecida, whereas a simple U-shaped digestive tract is a trait of the Hyolithida. We assume that the type 3 digestive tubes developed later during ontogeny, so *C. subcurvata* exhibits a hyolithid organisation of digestive tract in its juvenile stages and acquires an orthothecid-type gut at later developmental stages. However, the largest specimens encountered in the Heraultia Limestone (possibly up to 2 mm in diameter, 1.2 mm in width, 4 mm in length in the largest confidently identified, almost complete specimens) are rare, generally not entirely preserved and lack intestines. The studied specimens (with preserved intestines) can be considered as small, even for micro-sized hyoliths (chemically extracted), because specimens of this species can reach up to 1 mm in diameter and 3.5 mm in length [64]. Size-related preservation of the gut was noticed in [42] and interpreted as reflecting the absence of a retractable operculum in larger stages in observed taxa (gut-bearing specimens were wrongly assigned to three different taxa in [42]); this would disfavour such preservation in comparison with the total assemblage, in which many preserved specimens attain and even exceed 1 cm in length. This surprising conclusion diverges from other observations of adults from different orthothecid taxa with a preserved retractable operculum [14], [23], [31], [63]. Moreover, extensive study of the Heraultia Limestone [41] invalidates this assumption as such large specimens are either very rare (and generally unidentifiable) or absent from the formation and would favour a general taphonomic bias in the size of preserved assemblage. Therefore, it is concluded that the ontogenic trend observed herein represents only the relatively early stages of ontogeny. It might be assumed that the folding of the intestine continued during later (unknown) stages, to reach the typical orthothecid configuration in larger specimens (specimens with intestine described in [33], all broken, have a minimum length of 4 cm and the complete specimen figured in [31] is 14,5 mm long). The hyolithid specimen figured in [30], with the typical simple U gut, is about 10 mm long, so it can be considered also at a later ontogenetic stage than the specimens studied herein.

**Figure 10:**
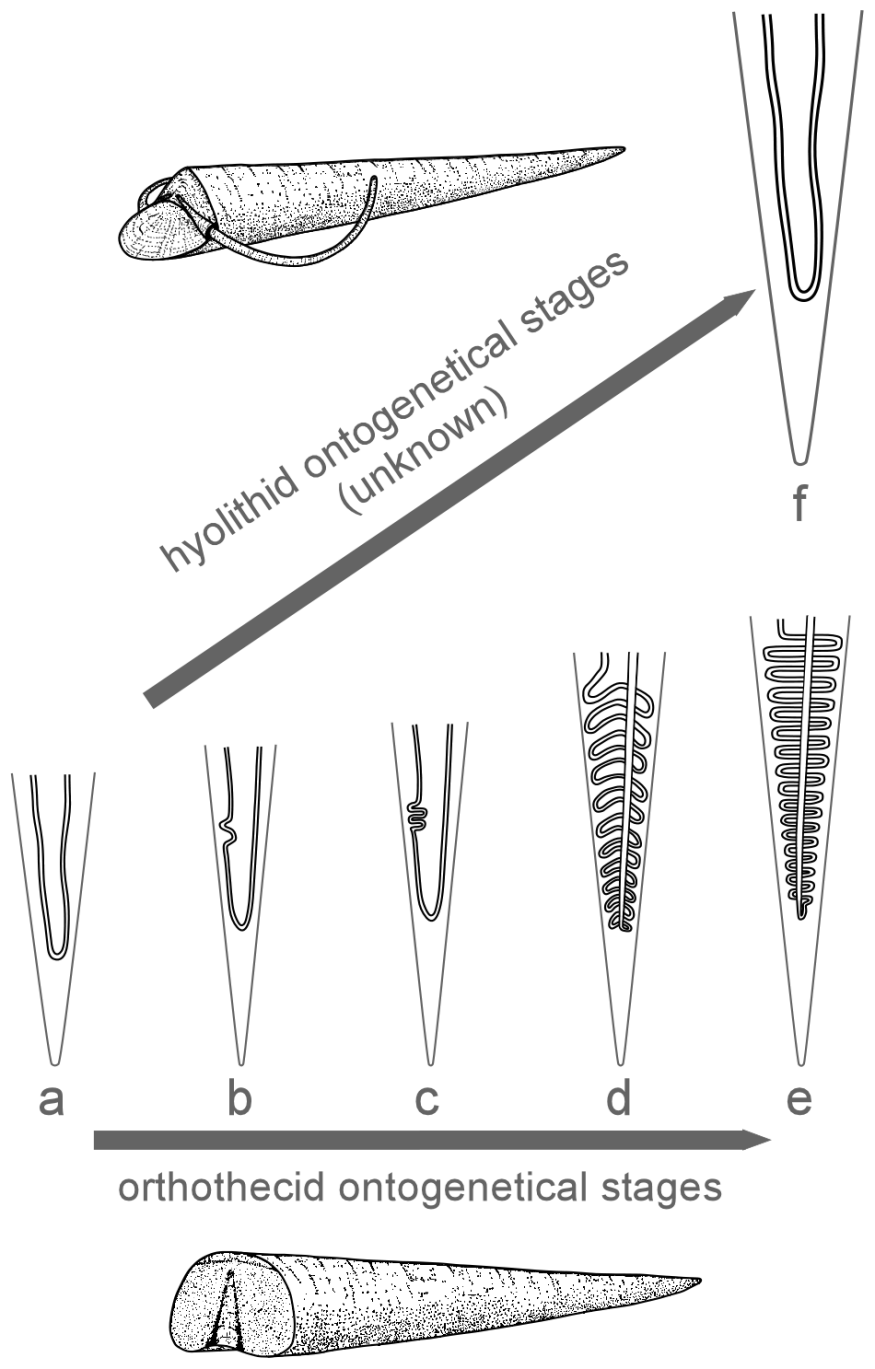
Illustration of hypothetised heterochronic processes of intestine evolution in hyoliths. a-c, based on the new data from the digestive tracts of *C. subcurvata*; d, from digestive tracts of *Guduguwan* from [9]; e, *Orthotheca* from [31]; f, *Haplophrentis* from [29], [30], [35], [36].

In summary, as stated by most authors, we consider that Orthothecida and Hyolithida were phylogenetically related, but we further suggest that the hyolith intestine evolved through heterochonic processes. The earliest occurrence of orthothecids slightly predates the first known hyolithids [65] in the early Cambrian, suggesting that the simple U-shaped gut in the hyolithids may have evolved through paedomorphosis from the orthothecid juvenile condition (Figure 10). This would be supported by the recognition that juvenile growth stages in *C. subcurvata* are characterised by a simple U-shaped digestive tract typical of Hyolithida whereas larger specimens exhibit one straight anal tube, the gut being tightly sinuously-folded in a small portion, a pattern further developed in larger specimens of Orthothecida through the entire length of the gut. This implies a primitive deposit-feeding strategy in hyoliths, and of orthothecids, which has previously been suggested (e.g. [9], [66]). However, the discovery of earlier hyolithids would question a paedomorphic development of hyolithids but argues for a peramorphosis of orthothecids.

Interpretation of suspension-feeding as the primitive and juvenile feeding strategy invokes such peramorphic processes [42]. In this model, the lengthening of the gut within the orthothecid lineage is viewed as a change of feeding strategy from suspension-feeding (getting the nutrient from a highly energetic source) to a deposit-feeding (depending on a less energetic source). Such an evolutionary pattern in gut configuration, linked to adaptation to different feeding strategies, has also been demonstrated among different arthropod lineages (e.g., from deposit to predatory feeding, see [58] or [67]), although this plasticity in gut-configuration in different lineages is unlikely to be ascribed to heterochrony. However, the adaptation of gut-configuration occurred only once among hyoliths, marked the divergence of the two hyolith lineages, and corresponds to either a reduction or addition of ontogenetic stages as shown herein. It is therefore better interpreted as reflecting heterochrony. In addition, the terrigenous gut-content and taphonomic features of the simplest U-gut in most juvenile specimens recovered herein are identical to the latest folded gut associated with largest conchs and argue for a deposit-feeding strategy. Even if these might not represent the earliest stages in the orthothecid ontogeny, they suggest that the modification of gut-architecture cannot be directly correlated with a shift in feeding strategy. The peramorphosis hypothesis involves speculation without supporting evidence; deposit-feeding is the favoured primitive feeding strategy among hyoliths.

J to U-shaped digestive tracts generally occur in organisms whose viscera are enclosed in blind tubes (ectoprocts) and burrows (sipunculans, phoronids), shells (gastropods, brachiopods) or calices (entoprocts). In gastropods, the gut, like the whole body-plan, undergoes torsion. Such a configuration cannot be directly related to the feeding behaviour: gastropod molluscs exhibit all possible feeding strategies. Brachiopods, phoronids, entoprocts and ectoprocts are all suspension-feeders. Even if the external-anatomy of their digestive tract is broadly similar to these suspension-feeders, although better described as J-shaped, sipunculan species differ in the way they obtain food. They can be suspension-feeders but most of them are deposit-feeders (i.e., scrape food or prey from the substrate surface). These observations suggest that the general organisation of the intestine cannot be interpreted directly in terms of feeding behaviour, but is instead mostly influenced by the sedentary mode of life, by constraints provided by the shape of the external skeleton, or, possibly, by the size of organism and energetic needs. Preserved intestine-content, although exceptional, constitutes the best evidence to identify the diet of extinct, basal taxa [68]. The increase of relative gut length through folding as observed herein may be better interpreted as either an adaptation to lower energy content in food-items or, as favoured herein, an evolution to a more efficient energy-capture by volume of ingested food without change of food items. Whatever the process involved in the described evolution, it seems obvious that it was most probably driven by ecological constraints.

Although J to U-shaped digestive tracts are common among modern taxa, they generally differ from the pattern described in orthothecids in a coiling and twisting of the intestine and a more or less complex structure with differentiated parts involving pouches, glands or caeca. In gastropods and brachiopods, an inflated stomach is clearly differentiated from the neighbouring oesophagus or pharynx and intestine. In ectoprocts, a muscular pharynx, a three-part stomach, associated with a caecum and finally a straight intestine leading to the rectum and anus are distinct. In the tubular phoronids, the U-shaped digestive tract is simple, without any coiling or folding, similarly to what can be observed in smallest specimen described in the present study. However, in Phoronids, oesophagus, stomach and intestine are differentiated to some extent, in contrast to hyoliths. Finally, the suspension-feeding entoprocts, which are organised into a stalk bearing a tentaculate calyx, possess a U-shaped intestine with an inflated stomach. Cambrian representatives of this group have been reported from the Chengjiang Biota [69] in which such an organisation of the digestive tract has already been observed.

Rather, the digestive tract of *C. subcurvata* is better compared to that of sipunculans, which are non-mineralised, non-segmented coelomate worms, mostly living in blind tubes or abandoned shells. Their body is arranged as a slender anterior section (introvert) that can be fully retracted into the posterior section (trunk). Sipunculans have a J-shaped gut, with the anus located close to the mouth when the introvert is retracted. In modern taxa, the descending and ascending halves of the recurved gut are both twisted and intertwined into a double helix or, in some taxa, folded into more irregular series of looser loops and partial coils (*Phascolion*; [70]). The number of gut coils increase with age and is not species-dependent. There is no clear external demarcation between the functional regions: the oesophagus and rectum are straight and the stomach is only slightly differentiated in three modern species [70]. This sipunculan configuration of the digestive tract exhibits clear similarities with *C. subcurvata*. Affinities between sipunculans and hyoliths have even been proposed [11], [71]. However, the two parts of the gut in *C. subcurvata* and other orthothecids with preserved gut (see list above), are not helically coiled as in modern sipunculans but folded, and the ‘ascending branch’ (anal tube) is straight. Nevertheless, in fossil sipunculans recovered in the early Cambrian Maotianshan Shale of China by [72], the J-shaped gut is composed of two straight descending and ascending halves (*Cambrosipunculus tentaculatus*) or straight descending and loosely folded ascending halves (*Archaeogolfingia caudata*; [72]). This configuration is closer to that of *C. subcurvata*. In modern sipunculans, stomach is defined based on its epithelium which glands release first acids and then enzymes that activate at the end of the descending half of the tract and the beginning of the ascending one [70]. All absorption takes place in the ascending intestine [70]. Most modern sipunculans are deposit-feeders that non-selectively collect, with their tentacles, sand, detritus, diatoms, and smaller invertebrates that fall into their burrow or use their introvert to explore the surrounding sediment [70]. Analysis of the gut content of modern representatives of the group showed that the sand in the gut is granulometrically similar to that of the surroundings [73]. The intestine of the Cambrian specimens, described by [72], is partly filled with sediment and this is interpreted by the authors as evidence that these animals collected organic material from the surrounding sediment surface like their modern representatives. Similarities in gut configuration and feeding strategy with sipunculans further allow extrapolation of functional anatomy of the digestive tract in *C. subcurvata.* The linear ingoing part of the gut located before the folded portion in *C. subcurvata* might have worked as the oesophagus of sipunculans while the next folded portion might have functioned as a stomach and absorption organ. Even if absorption might have also occurred in the linear anal tube, comparison with sipunculans would question any significant function of this portion of the gut. It is possible, also by comparison with gastropods, that this part was mostly involved in transformation and storage of faecal pellets. The increase in volume of a conical-body (based on diameter and height) is exponential, while the growth of a simple U-shaped gut would be linear (for a constant diameter). The increase in number of gut-folds during individual growth would have then increased the length of secretion (digestion) and absorption to meet a higher energy requirement of adult organisms. Moreover, gut/body ratio (absorption rate per volume) is considered to be higher in deposit-feeders (as suggested by [74]).

## Conclusion

Three-dimensionally preserved, phosphatised, digestive tracts are reported in orthothecids from the Cambrian (Terreneuvian) of Northern Montagne Noire (France). The smooth, uniseriate, branching to anastomosing filaments, along with isolated botryoidal coccoids, observed at the surface of the U-shaped digestive tracts attest to a microbially mediated phosphatisation. Moreover, cryptic microbes mediated the early phosphatisation of the gut content prior to decay of muscles and other tissues which supported the digestive tracts. The digestive tract content (mainly phosphate but also minor terrigenous and bioclastic material) has been analysed and supports a deposit-feeding strategy.

These phosphatised digestive tracts in orthothecids from the Montagne Noire help to clarify the relationships between orthothecids and hyolithids, supporting the notion that Orthothecida and Hyolithida might have been related phylogenetically by heterochronic processes. The different hyolith gut-types may represent a heterochronic development reflecting possible adaptation to different feeding strategies.
